# The association between gut microbiota and postoperative delirium in patients

**DOI:** 10.1038/s41398-023-02450-1

**Published:** 2023-05-09

**Authors:** Yiying Zhang, Kathryn Baldyga, Yuanlin Dong, Wenyu Song, Mirella Villanueva, Hao Deng, Ariel Mueller, Timothy T. Houle, Edward R. Marcantonio, Zhongcong Xie

**Affiliations:** 1 Geriatric Anesthesia Research Unit, Department of Anesthesia, Critical Care and Pain Medicine, Massachusetts General Hospital, Harvard Medical School, Charlestown, MA 02129 USA; 2 Department of Medicine, Brigham and Women’s Hospital, Harvard Medical School, Boston, MA 02115 USA; 3 Department of Anesthesia, Critical Care and Pain Medicine, Massachusetts General Hospital, Harvard Medical School, Boston, MA 02114 USA; 4 Divisions of General Medicine and Gerontology, Department of Medicine, Beth Israel Deaconess Medical Center, Harvard Medical School, Boston, MA 02215 USA

**Keywords:** Predictive markers, Molecular neuroscience, Long-term memory

## Abstract

Postoperative delirium is a common postoperative complication in older patients, and its pathogenesis and biomarkers remain largely undetermined. The gut microbiota has been shown to regulate brain function, and therefore, it is vital to explore the association between gut microbiota and postoperative delirium. Of 220 patients (65 years old or older) who had a knee replacement, hip replacement, or laminectomy under general or spinal anesthesia, 86 participants were included in the data analysis. The incidence (primary outcome) and severity of postoperative delirium were assessed for two days. Fecal swabs were collected from participants immediately after surgery. The 16S rRNA gene sequencing was used to assess gut microbiota. Principal component analyses along with a literature review were used to identify plausible gut microbiota, and three gut bacteria were further studied for their associations with postoperative delirium. Of the 86 participants [age 71.0 (69.0–76.0, 25–75% percentile of quartile), 53% female], 10 (12%) developed postoperative delirium. Postoperative gut bacteria *Parabacteroides distasonis* was associated with postoperative delirium after adjusting for age and sex (Odds Ratio [OR] 2.13, 95% Confidence Interval (CI): 1.09–4.17, *P* = 0.026). The association between delirium and both *Prevotella* (OR: 0.59, 95% CI: 0.33–1.04, *P* = 0.067) and *Collinsella* (OR: 0.57, 95% CI: 0.27–1.24, *P* = 0.158) did not meet statistical significance. These findings suggest that there may be an association between postoperative gut microbiota, specifically *Parabacteroides distasonis*, and postoperative delirium. However, further research is needed to confirm these findings and better understand the gut-brain axis’s role in postoperative outcomes.

## Introduction

Postoperative delirium one of the most common postoperative complications in older patients [1], and is associated with nosocomial complications [2]; extended hospital stays [3], a higher chance of institutional discharge [4, 5], and increased morbidity [5–8] and mortality [9, 10]. One study has estimated the annual healthcare costs in the United States attributable to postoperative delirium to be $32.9 billion [11]. Despite its clinical importance, there are currently no targeted interventions for postoperative delirium due to an incomplete understanding of its pathogenesis.

Inflammation, neuroinflammation, and Alzheimer’s disease neuropathogenesis (e.g., phosphorylated tau) have been reported as biomarkers and pathogenesis of postoperative delirium [1, 12, 13]. However, identifying the causes, contributing factors, pathogenesis, and biomarkers of postoperative delirium remains to be fully elucidated, hindering progress in the field.

The gut microbiota constitutes up to 95% of the total human microbiota [14], and it is widely recognized that the gut-brain axis plays an essential role in regulating brain function [15–18]. Dysregulation of the gut microbiota has been associated with alterations in immune function and increased risk of certain diseases [19]. Specifically, gut microbiota dysbiosis, an imbalance of gut microbiota associated with adverse outcomes, has been linked to cognitive impairment and Alzheimer’s disease neuropathogenesis [14, 20–23].

In a previous study of 18-month-old mice, anesthesia and surgery were associated with changes in gut microbiota and cognitive impairment 48 h and 5–8 days after the anesthesia/surgery, respectively [24]. A recent study showed that gut microbiota alteration contributes to developing postoperative cognitive impairment in mice [25]. Our recent animal study showed that anesthesia/surgery reduced the abundance of gut lactobacillus and induced delirium-like behavior in the 18-month-old mice [26]. Treatment with lactobacillus or probiotics mitigated the anesthesia/surgery-induced delirium-like behavior in the mice [26]. These pre-clinical data suggest that gut microbiota may contribute to the pathogenesis and serve as a biomarker of postoperative delirium. However, to our knowledge, no clinical studies have shown the association between gut microbiota and postoperative delirium in patients.

This prospective observational cohort study aimed to investigate the association between changes in postoperative gut microbiota and postoperative delirium in patients. Specifically, we aimed to test the hypothesis that alterations in gut microbiota may be associated with an increased risk of postoperative delirium. The findings of this study could advance our understanding of the risk factors, biomarkers, pathogenesis, and potential interventions for postoperative delirium in patients. They may encourage further research in this area.

## Methods

### Study enrollment

Upon approval by the Mass General Brigham Institutional Review Board, this prospective observational cohort study was performed at Massachusetts General Hospital, Boston, MA, between 2016 and 2020. Patients 65 years or older, proficient in English, and scheduled for elective knee replacement, hip replacement, or laminectomy under general or spinal anesthesia at the study hospital were included.

Patients were excluded from participation if they had any of the followings: (1) past medical history of neurological and psychiatric diseases, including Alzheimer’s disease (AD), other forms of dementia, stroke, or psychosis; (2) severe visual or hearing impairments; (3) current smokers; or (4) taking antibiotics within one week of surgery. Trained clinical research coordinators approached eligible patients for participation during preoperative clinic visits. Written informed consent was obtained at the time of enrollment, prior to the initiation of the study procedures. This manuscript is being reported following the STrengthening the Reporting of OBservational studies in Epidemiology (STROBE) criteria.

### Anesthesia, surgery, and fecal sample collection and measurement

Enrolled participants had a knee replacement, hip replacement, or repair of spinal stenosis under general or spinal anesthesia. All participants received standardized perioperative care, including standard postoperative pain management (e.g., patient-controlled analgesia with hydromorphone). Depth of sedation was at the discretion of the treating provider but was not captured in the current study. There have been no significant changes in the surgery or anesthesia practice since the start of the study. Fecal swabs were collected from participants immediately after surgery. The 16S rRNA gene sequencing was performed by BGI America (Cambridge, MA) as previously described [26]. The relative abundance in 16S rRNA sequencing refers to the proportion of a specific bacterial species or group of bacteria relative to the total number of bacterial sequences in each sample. In this study, we employed relative abundance values to compare the bacterial composition between different samples or groups.

### Determination of postoperative delirium

Trained clinical research coordinators interviewed participants to determine the presence or absence of postoperative delirium on postoperative days one and two when applicable. The Confusion Assessment Measurement (CAM) was used in this study as a diagnostic algorithm to determine the presence or absence of delirium, which has high reliability [27, 28]. The incidence of postoperative delirium, the primary outcome, was assessed using the CAM assessment once per day between 8:00 a.m. and 12:00 noon. Sixty-four of the 86 participants were evaluated on both days. One of the 10 participants with postoperative delirium and 21 of the 76 participants without postoperative delirium had the CAM only on one day. Delirium was defined as present if it occurred on either postoperative day one or day two. The preoperative CAM was not performed in the present study because previous studies [29, 30] have shown that participants who underwent elective surgeries had a very low incidence of preoperative delirium.

The secondary outcome was the severity of postoperative delirium, represented by the Memorial Delirium Assessment Scale (MDAS) [28, 31], quantifying delirium-related symptoms based on 10 features. Each feature is scored from 0 (best) to 3 (worst symptom) with a maximal score of 30. We determined the MDAS scores for all patients, regardless of whether they had the presence of delirium, based on the results from CAM, on that day. The average MDAS score (averaged from both days if two postoperative tests were performed or from one day if only one postoperative test was performed) or peak MDAS score was used to assess delirium severity independent of the results obtained from CAM. We performed the postoperative MMSE as part of CAM [27, 32] and also for MDAS calculation on postoperative day one and day two [32].

### Dimension-reduction algorithm (DASH)

We developed a specialized feature engineering process to reduce the complexity of input variables without losing important information. Specifically, principal component analysis (PCA) was performed among 740 gut bacteria to reduce these variables’ dimensions (one variable per gut bacteria). The eigenvalue above the 3.0 PC component was chosen, representing 72.8% of the total variance in the dataset. Second, bacteria from the selected PC component were ranked according to the contribution index from highest score (e.g., ±0.12) to lowest score (e.g., ± 0.003), resulting in the top 35 gut bacteria. Third, gut bacteria without information at the species level were excluded, resulting in 15 gut bacteria. Fourth, eight gut bacteria were selected from the 15 gut bacteria based on domain expertise, showcasing that these eight bacteria were associated with human diseases from previous studies. Finally, three gut bacteria (*Parabacteroides distasonis, Prevotella*, and *Collinsella*), excluding the other five gut bacteria, were investigated in the present study based on their relevance with inflammation, which contributes to cognitive dysfunction [33–38].

### Statistical analysis

Descriptive statistics were conducted using methods appropriate for the variables under this study. Means and standard deviations were used for continuously scaled variables that were normally distributed. Medians and 25th and 75th percentiles were used for skewed or ordinal data. Frequency counts and percentages or proportions were used for categorical variables. Differences in baseline characteristics between those who did and did not develop delirium were assessed with a *t*-test, Mann–Whitney *U* test (for non-normal continuous data), chi-square, or Fisher’s exact test (in the case of small cell counts), as appropriate.

A principal component analysis (PCA) was conducted to reveal hidden patterns across high-dimensional bacteria abundance measurements at the species level. Robust logistic regression was then used to assess the relationship between the three selected gut bacteria and postoperative delirium as the binary outcome. Results were presented as odds ratio (OR) per one unit of relative abundance of gut bacteria change in the biomarker and their associated 95% confidence intervals (CI). Linear regression was used to evaluate the association between bacteria and delirium severity (using both average and peak MDAS scores) as the continuous outcome, with results presented as a mean difference (beta coefficient [*β*]) and its associated 95% confidence interval. Models were created to adjust for the associations between the biomarkers and outcomes for age and sex for both the primary and secondary outcomes. Variables for adjustment were based on previous studies as deemed clinically relevant.

The present study did not consider multiple comparisons because of the exploratory nature in which the final association model was based, reduced inputs with a small number (e.g., three bacteria) of independent variables according to the statistical principles described before [39]. All analyses were conducted using R version 4.0.5 statistical software (Vienna, Austria). All analyses used two-tailed hypothesis testing where appropriate, with statistical significance interpreted at *p* < 0.05.

### Reporting summary

The paper’s raw sequence data has been deposited in the Genome Sequence Archive and is publicly accessible at https://www.ncbi.nlm.nih.gov/sra/PRJNA967718. The accession number for the data has been deposited in accordance with the guidelines set out by Genomics, Proteomics & Bioinformatics in 2021 and Nucleic Acids Res in 2022. This information is essential for anyone wishing to access or use the data for further research or analysis. Simplesubmission.com performed the submission of the raw sequence data.

## Results

**Fig. 1:**
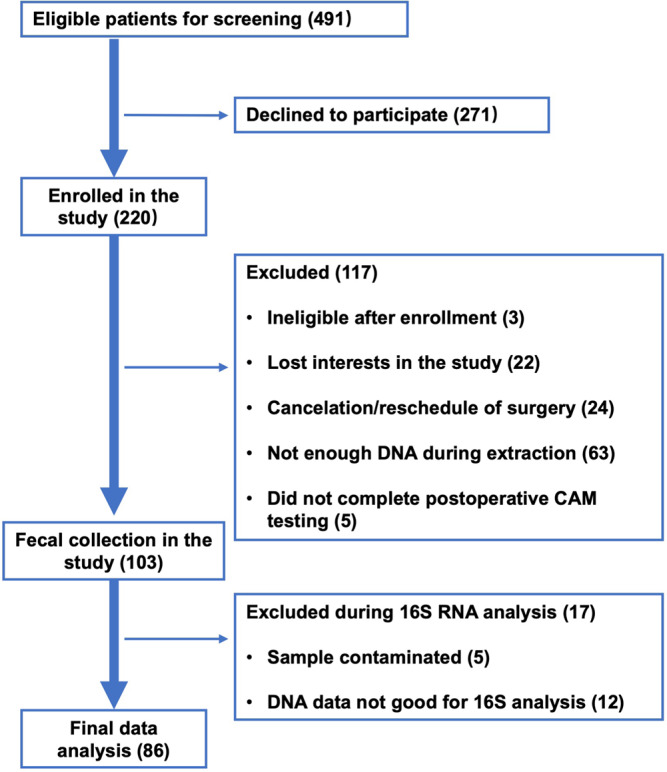
Flow diagram. The flow diagram shows that 491 participants were screened for the studies, and 220 were initially enrolled. One hundred seventeen participants were excluded after enrollment, and 103 were included in the gut microbiota cohort. During the analysis, 17 additional participants were excluded, resulting in 86 participants for the final data analysis.

A total of 491 patients were screened, of which 220 participants were enrolled. A total of 117 participants were excluded owing to becoming ineligible after enrollment (*N* = 3), no longer expressing interest in participating (*N* = 22), cancellation/rescheduling surgery (*N* = 24), not having enough DNA during extraction (*N* = 63), or not completing the postoperative CAM testing (*N* = 5). Thus, 103 participants were included in the gut microbiota cohort. Of these, 17 participants were further excluded due to DNA sample contamination (*N* = 5) and poor quality of 16S rRNA gene sequencing (*N* = 12). Thus, 86 participants were included in the data analysis (Fig. 1). There were no significant differences in the demographic characteristics between the participants included (*N* = 86) and those excluded (*N* = 134; Supplemental Table 1). There were no significant complications among participants during the immediate postoperative period.

Ten of the 86 (12%) participants developed postoperative delirium (Table 1). The baseline demographic and clinical characteristics of the 86 participants were presented in Table 1. There were no significant differences in age, gender, ethnicity, surgery type, anesthesia type, or preoperative Mini-Mental State Examination (MMSE) score between the participants with postoperative delirium (*N* = 10) and those without postoperative delirium (*N* = 76). The participants who developed postoperative delirium had lower postoperative MMSE scores and higher MDAS scores than the participants who did not develop postoperative delirium (Table 1).

**Table 1:** Demographic characteristics of the participants.

	Delirium (*N* = 10)	No Delirium (*N* = 76)	*P* value
Age, median (25–75% percentile of quartile)	72.0 (70.5–75.3)	71.0 (69.0–76.8)	0.499
Female, *n* (%)	4 (40)	42 (46)	0.521
Non-white or Hispanic, *n* (%)	1 (10)	3 (3.9)	0.289
Education years, median (25–75% percentile of quartile)	16 (16.0–16.0)	16.4 (16.0–18.0)	0.866
Surgery type, *n* (%)			
Knee replacement	5 (50)	48 (63)	0.332
Hip replacement	3 (30)	23 (30)	
Spinal stenosis	2 (20)	5 (7)	
Anesthesia type, *n* (%)			
General	6 (60)	40 (53)	0.661
Spinal	4 (40)	36 (47)	
MMSE, median (25–75% percentile of quartile)			
Pre-surgery score	29.0 (29.0–30.0)	29.0 (28.0–30.0)	0.734
Post-surgery score	27.5 (26.5–28.6)	29.0 (28.5–30.0)	0.004
MDAS score (average) mean ± SD	5.15 ± 1.53	1.98 ± 1.39	<0.01
MDAS score (peak) mean ± SD	6.60 ± 1.78	2.45 ± 1.64	<0.01

*MMSE* mini-mental status examination, *MDAS* Memorial Delirium Assessment Scale, *SD* standard deviation.

### Postoperative gut microbiota abundances were associated with the incidence of postoperative delirium

A total of 740 postoperative gut bacteria were identified and used in the principal component analysis (PCA). The participants with and without postoperative delirium showed a significant difference in the index of principal component 8 (Supplementary Fig. 1). Using the methods described above, three gut bacteria were identified, with the association between these bacteria and the incidence and severity of delirium presented in Table 2. In unadjusted analyses, the abundance of postoperative gut bacteria *Parabacteroides distasonis* was associated with postoperative delirium (OR 1.97, 95% CI: 1.04–3.74, *P* = 0.038). There was no statistically significant association between the abundances of *Prevotella* (OR 0.62, 95% CI: 0.36–1.07, *P* = 0.085) or *Collinsella* (OR 0.60, 95% CI: 0.28–1.30, *P* = 0.197) and postoperative delirium (Table 2).

**Table 2:** Association between gut bacteria and postoperative delirium^a^.

Presence of postoperative delirium^b^				
	Unadjusted		Adjusted for age and sex	
	Odds ratio (95%CI)	*P* value	Odds ratio (95% CI)	*P* value
*Parabacteroides distasonis*	1.97 (1.04–3.74)	0.038	2.13 (1.09–4.17)	0.026
*Prevotella*	0.62 (0.36–1.07)	0.085	0.59 (0.33–1.04)	0.067
*Collinsella*	0.60 (0.28–1.30)	0.197	0.57 (0.27–1.24)	0.158

The severity of postoperative delirium^c^				
	Unadjusted		Adjusted for age and sex	
	*β* coefficient (95% CI)	*P* value	*β* coefficient (95% CI)	*P* value
*Parabacteroides distasonis*	0.23 (−0.06 to 0.52)	0.123	0.21 (−0.13 to 0.55)	0.226
*Prevotella*	−0.12 (−0.32 to 0.08)	0.242	−0.11 (−0.32 to 0.09)	0.288
*Collinsella*	−0.14 (−0.45 to 0.17)	0.383	−0.10 (−0.45 to 0.24)	0.564

^a^Models were created to adjust the associations between the bacteria and outcomes for age and sex based on previous studies as deemed clinically relevant.

^b^Results are presented as odds ratio (OR) per one unit change in gut bacteria value and its associated 95% confidence intervals (CI).

^c^Results are presented as the beta coefficient (*β*) per one unit change in gut bacteria value and its associated 95% CI.

After the adjustment for age and sex, similar results were observed. The abundance of postoperative *Parabacteroides distasonis* was significantly associated with postoperative delirium (OR 2.13, 95% CI: 1.09–4.17, *P* = 0.026). *Prevotella* (OR: 0.59, 95% CI: 0.33–1.04, *P* = 0.067) and *Collinsella* (OR 0.57, 95% CI: 0.27–1.24, *P* = 0.158) were not significantly associated with the incidence of postoperative delirium. Further, the abundance of postoperative *Parabacteroides distasonis*, *Prevotella* and *Collinsella* were not associated with the severity of postoperative delirium, both before and after adjusting for age and sex (Table 2).

### Postoperative gut microbiota abundances were different between the participants with and without postoperative delirium

**Fig. 2:**
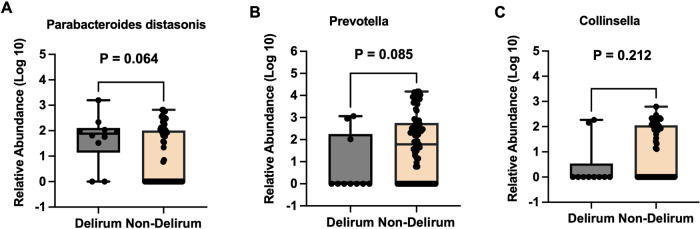
Different postoperative gut bacteria between participants with and without postoperative delirium. Participants who developed postoperative delirium (*N* = 10) had a higher postoperative relative abundance of gut bacteria of *Parabacteroides distasonis* (**A**) a lower abundance of postoperative gut bacteria of *Prevotella* (**B**) but not *Collinsella* (**C**) than the participants who did not develop postoperative delirium (*N* = 76). The box indicates the median (50th percentile), the first quartile (25th percentile), and the third quartile (75th percentile) of the abundance of bacteria. Mann–Whitney *U* test was used to determine the differences in bacteria abundance between the participants with postoperative delirium and those without postoperative delirium.

Our analysis revealed that participants who developed postoperative delirium had higher *Parabacteroides distasonis* in their postoperative gut microbiota (average abundance 1.659 ± 0.984) compared to those who did not develop delirium (average abundance 0.850 ± 1.080) although, this difference was only marginally significant (Mann–Whitney *U* test, *P* = 0.064, Fig. 2A). Additionally, we observed lower levels of postoperative gut *Prevotella* in participants with postoperative delirium (average abundance 0.803 ± 1.321) compared to those without postoperative delirium (average abundance 1.687 ± 1.459), again making this difference only marginally significant (Mann–Whitney *U* test, *P* = 0.085, Fig. 2B). However, we did not observe any significant difference in postoperative gut *Collinsella* abundance between the two groups (average abundance 0.442 ± 0.932 vs. 0.903 ± 1.040, Mann–Whitney *U* test, *P* = 0.212, Fig. 2C).

## Discussion

In this prospective observational cohort study, we observed an association between the gut bacteria *Parabacteroides distasonis* and the incidence of postoperative delirium in patients. These findings suggest that gut microbiota dysbiosis, linked to several brain dysfunctions [15–18], may play a role in the development of postoperative delirium. Further studies are needed to confirm these results and explore the potential of gut bacteria as biomarkers, pathogenesis, and intervention targets for postoperative delirium.

In the present study, the incidence of postoperative delirium was 12%, consistent with the incidence rates reported in other clinical investigations of postoperative delirium [40–43] that have reported rates ranging from 5.1% to 19.4% in patients. The characteristics of the participants, including their average and peak MDAS scores, are presented in Table 1. Future studies will include a table that lists the characteristics of participants according to various abundances of the gut microbiota.

Increases in the abundance of postoperative gut bacteria *Parabacteroides distasonis* were associated with an increased incidence of postoperative delirium in the patients after adjusting age and sex. Although generally lower in delirious patients, decreases in the abundance of *Prevotella* and *Collinsella* were not significantly associated with delirium. Moreover, patients who developed postoperative delirium had a higher abundance of postoperative gut *Parabacteroides distasonis* and a lower abundance of postoperative gut *Prevotella* and *Collinsella*. These data suggest the potential contribution of the gut-brain axis to postoperative delirium, and certain postoperative gut bacteria that are associated with postoperative delirium.

The objective of the present study was to investigate the potential association between postoperative gut microbiota and postoperative delirium in patients. We used postoperative samples in this proof-of-concept study to establish a system for determining whether postoperative gut microbiota may contribute to the development of postoperative delirium in patients.

*Parabacteroides distasonis* is a bacteria implicated in Crohn’s Disease, ulcerative colitis [36] and *Prevotella* is associated with chronic inflammatory disease [37]. Therefore, future studies should investigate the potential association between postoperative delirium and Crohn’s Disease, ulcerative colitis, and chronic inflammatory diseases. In addition to inflammation, *Parabacteroides distasonis* may influence postoperative delirium by generating metabolites that could directly impact brain function. For instance, some gut bacteria, such as those producing short-chain fatty acids (SCFAs), have been shown to affect brain function and behavior, and *Parabacteroides distasonis* may produce SCFAs or other metabolites with similar effects. Moreover, changes in the gut microbiome’s composition, such as changes in the abundance of *Parabacteroides distasonis*, may have downstream effects on other gut bacteria, which could affect brain function. Future studies to test this hypothesis are warranted.

Previous studies have shown that patients who developed postoperative delirium (*N* = 20) and who did not develop postoperative delirium (*N* = 20) had a different abundance of preoperative gut bacteria [44]. Specifically, gut bacteria *Proteobacteria, Enterobacteriaceae, Escherichia shigella, Klebsiella, Ruminococcus, Roseburia, Blautia, Holdemanella, Anaerostipes, Burkholderiaceae, Peptococcus, Lactobacillus*, and *Dorea* were abundant in the patients with postoperative delirium, and *Streptococcus equinus* and *Blautia hominis* were abundant in the patients without postoperative delirium [44]. However, this previous study is different from the current study, as it determined preoperative, not postoperative, gut microbiota, did not establish an association with the incidence of postoperative delirium, and did not assess the severity of delirium with the MDAS. Thus, the previous study did not demonstrate the association between gut microbiota and postoperative delirium in patients. Future studies should include the systematical determination of the association between postoperative delirium and both pre-and postoperative gut microbiota in a larger-scale study.

Interestingly, the present study did not find associations between the three gut bacteria and the severity of postoperative delirium in patients, as represented by average MDAS scores (Table 2) or peak MDAS scores (data not shown). However, a previous study also indicates that some biomarkers are only associated with the incidence, not severity, of postoperative delirium in patients [45].

Notably, the average and peak MDAS scores of the present study participants were 5.15 and 6.60, respectively (Table 1). Although Breitbart et al. stated that MDAS Scores ≥13 indicate the presence of delirium [31], the study’s participants included psychiatry consult patients [31]. Marcantonio et al. showed that the best MDAS cutoff for postoperative delirium was 5 in the participants with surgery for hip fracture repair [28]. Therefore, it is reasonable that the average MDAS score was 5.15 (average) and 6.60 (peak) in the present study.

One strength of our study was the use of a Dimension-reduction Algorithm in Small Human-datasets (DASH) that combined statistical dimensionality reduction algorithms and domain expertise to efficiently extract accurate signals from the noise background in high-throughput data screening. Given the small sample size and complex data structure, data-driven methodology alone was insufficient in finding the relationship between gut microbiota and postoperative delirium in patients. By incorporating our current knowledge into the data-driven methodology, we could filter through several hundred variables in a small dataset, making this method particularly powerful for analyzing small but high-dimensional patient-level datasets. However, it should be noted that the selection strategy used with principal component analysis (PCA), such as excluding bacteria without species, could result in selection bias in the present study. Nevertheless, in the present study, we identified *Parabacteroides distasonis* because it is associated with inflammation-related disorders [36], and inflammation is associated with postoperative delirium [12]. *Prevotella* was selected because of its association with chronic inflammatory disease [37], and *Collinsella* was chosen because of its known association with cumulative inflammatory response [38].

This study had limitations, including a small sample size from a single center and a low number (10) of participants who developed postoperative delirium. However, similar studies with smaller sample sizes (*N* = 11 [46] and *N* = 14 [47]) have drawn solid conclusions. Out of 220 enrolled participants, 134 were excluded mainly due to insufficient DNA amounts in the swapped samples. There were no significant differences in the characteristics between the 86 participants in the final data analysis and the 134 excluded patients, except for anesthesia type (Supplemental Table 1). However, previous studies have shown that anesthesia type does not affect the incidence of postoperative delirium [40, 48]. In addition, we did not perform preoperative CAM in the participants since these participants had elective cases and the rate of preoperative delirium would be very low based on the findings from previous studies by Mei et al. (0 of 606 participants) [29] and Shi et al. (3 of 192 participants) [30]. The presence of postoperative delirium in the present study may not be called incident delirium but rather postoperative delirium.

In conclusion, this proof-of-concept study established a potential link between postoperative gut bacteria *Parabacteroides distasonis* and postoperative delirium in patients. Patients who developed postoperative delirium had a higher abundance of this bacteria than those who did not. However, the association between postoperative gut microbiota and postoperative delirium was relatively weak in this study, and therefore, the clinical relevance of these findings needs further investigation in future research. Nevertheless, these findings suggest that gut microbiota dysbiosis may influence postoperative delirium, but more research is necessary to understand the role of gut microbiota in this condition.

## Supplementary information

Supplemental Information

## Data Availability

The source data can be available from the corresponding authors on reasonable request.
