# Supplementary material for: The association between gut microbiota and postoperative delirium in patients
Source: Transl Psychiatry. 2023 May 9;13:156. doi: 10.1038/s41398-023-02450-1 (PMC10170091; doi:10.1038/s41398-023-02450-1)
Supplement: Supplementary file 1 — Supplemental Information [file 41398_2023_2450_MOESM1_ESM.pdf]

Supplemental Information

The association between gut microbiota and postoperative delirium in patients

Yiying Zhang MD, PhD, Kathryn Baldyga BS, Yuanlin Dong MD MS,
Wenyu Song, PhD, Mirella Villanueva, M.S., Hao Deng, MPH , Ariel Mueller MA,
Timothy T Houle PhD, Edward R Marcantonio MD MS, Zhongcong Xie MD, PhD

Supplemntal Figure 1.

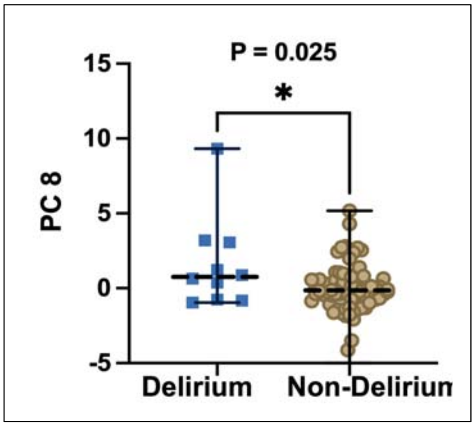

Supplemental Figure 1: Different postoperative gut bacteria profiles between participants with and without postoperative delirium.

The participants with postoperative delirium (left column) had different index of principal component 8 than the participants without delirium (right column). The box indicates median (50th percentile), the first quartile (25th percentile), and the third quartile (75th percentile) of the abundances of bacteria.

Supplemental Table 1. Demographic characteristics of the participants

	Included participants (N = 86)	Excluded participants (N = 134)	P-value
**Age, median (25%-75% percentile of quartile)**	**72.0 (69.0 – 76.0)**	**72.0 (68.0 – 76.0)**	**0.852**
**Female, n (%)**	**46 (53)**	**73 (54)**	**0.237**
**Non-white or Hispanic, n (%)**	**4 (5)**	**10 (7)**	**0.582**
**Surgery type, n (%)**			
**Knee replacement**	**53 (62)**	**96 (72)**	**0.251**
**Hip replacement**	**26 (30)**	**32 (24)**	
**Spinal stenosis**	**7 (7)**	**6 (4)**	
**Anesthesia type, n (%)**			
**General**	**46 (53)**	**52 (39)**	**0.038**
**Spinal**	**40 (47)**	**82 (61)**	
**MMSE, median (25% -75% percentile of quartile)**			
**Pre-surgery score**	**29.0 (28.8 – 30.0)**	**29.0 (28.0 – 30.0)**	**0.931**

MMSE, mini-mental status examination.
